# Recovery of Partially Engorged *Haemaphysalis longicornis* (Acari: Ixodidae) Ticks from Active Surveillance

**DOI:** 10.1093/jme/tjac099

**Published:** 2022-07-19

**Authors:** Keith J Price, Bryn J Witmier, Rebecca A Eckert, Christian N Boyer

**Affiliations:** Division of Vector Management, Pennsylvania Department of Environmental Protection, Harrisburg, PA 17110, USA; Division of Vector Management, Pennsylvania Department of Environmental Protection, Harrisburg, PA 17110, USA; Department of Environmental Studies, Gettysburg College, Gettysburg, PA 17325, USA; Division of Vector Management, Pennsylvania Department of Environmental Protection, Harrisburg, PA 17110, USA

**Keywords:** Scutal Index, blood feeding, Asian longhorned tick, blacklegged tick

## Abstract

The invasive Asian longhorned tick, *Haemaphysalis longicornis*, has rapidly spread across the northeastern United States and is associated with pathogens of public health and veterinary concern. Despite its importance in pathogen dynamics, *H. longicornis* blood-feeding behavior in nature, specifically the likelihood of interrupted feeding, remains poorly documented. Here, we report the recovery of partially engorged, questing *H. longicornis* from active tick surveillance in Pennsylvania. Significantly more engorged *H. longicornis* nymphs (1.54%) and adults (3.07%) were recovered compared to *Ixodes scapularis* nymphs (0.22%) and adults (zero). Mean Scutal Index difference between unengorged and engorged nymph specimens was 0.65 and 0.42 for *I. scapularis* and *H. longicornis*, respectively, suggesting the questing, engorged *H. longicornis* also engorged to a comparatively lesser extent. These data are among the first to document recovery of engorged, host-seeking *H. longicornis* ticks and provide initial evidence for interrupted feeding and repeated successful questing events bearing implications for pathogen transmission and warranting consideration in vector dynamics models.

The invasive Asian longhorned tick, *Haemaphysalis longicornis* Neumann (Acari: Ixodidae), has rapidly spread to 17, primarily northeastern, states since initial documentation of its establishment in the United States in 2017 (Rainey et al. 2018). In both its endemic and invaded ranges, *H. longicornis* can acquire and carry pathogens of human importance, including the agents causing Lyme disease and spotted fever group rickettsiosis (Qin et al. 2019, Zhao et al. 2020, Price et al. 2021a). Their broad host range coupled with habitat overlap and high prevalence of co-feeding with native vector tick species could facilitate pathogen acquisition and spillover, as recently described for arboviruses (Price et al. 2021b, Tufts et al. 2021, White et al. 2021, Cumbie et al. 2022). However, important factors in pathogen transmission risk, including blood-feeding behavior and engorgement dynamics, remain poorly documented in natural field conditions (Piesman 1993, Tahir et al. 2020). Therefore, our objective was to compare engorgement status of questing *H. longicornis* and other ticks recovered from active, statewide surveillance in Pennsylvania (PA).

## Methods

Pennsylvania Department of Environmental Protection conducts active tick surveillance targeting adult and nymphal *Ixodes scapularis* Say (Acari: Ixodidae) intermittently in all 67 counties from October through April (1 October 2020 to 8 April 2021) and weekly from May through August (1 May 2021 to 31 August 2021). Nontarget tick species, especially *H. longicornis*, are often collected as surveillance periods overlap with peak adult and nymphal activity (Supp Table 1 [online only]; Tufts et al. 2019, Piedmonte et al. 2021). Our sampling sites (*n*= 484) are largely public areas in deciduous forests and were selected for presence of suitable *I. scapularis* and reportedly suitable *H. longicornis* tick habitat (Tufts et al. 2019, Thompson et al. 2021). Field procedures followed Price et al. (2021b). Briefly, at each site questing ticks were collected by dragging a 1 m^2^ white felt cloth over vegetation and leaf litter for ≥100 m. The dragging method is broadly utilized for active surveillance of *I. scapularis* and preferred for collection of *H. longicornis* (Sherpa et al. 2021). Cloths were examined every 10 m and recovered ticks transferred into vials containing 80% ethanol; ticks were shipped to a central laboratory, identified to species using a Nikon SMZ-800N stereomicroscope and morphological keys (Keirans and Litwak 1989, Egizi et al. 2019), and classified by life stage, sex, and degree of engorgement (unengorged, partial, full).

Noticeably darkened abdomen and swollen opisthosoma served as criteria for distinguishing engorgement in all tick species (Nathanson 1970, Starck et al. 2018). Other diverged morphological features (e.g., narrowed subdivisions between festoons) were helpful for differentiating engorged *H. longicornis*. All engorged ticks as well as unengorged, field-collected reference specimens (*n* = 10/spp./life stage) were then measured for total body length and scutum length, the ratio of which functions as an engorgement (Scutal) index indicative of feeding duration (Yeh et al. 1995), using NIS-Elements software (Nikon Instruments Inc., Melville, NY; Supp Fig. 1 [online only]). Unfed *I. scapularis* obtained from a laboratory colony at the Oklahoma State University Tick Rearing Facility and *H. longicornis* obtained from a colony, established from a parthenogenic strain, at the Centers for Disease Control and Prevention were also measured to compare against unengorged, field-collected reference specimens. To confirm the presence of presumptive blood, a subset (*n* = 8) of engorged and unengorged nymphs were bisected on sterile microscope slides and swabbed with cotton applicators which were subjected to a phenolphthalein (Kastle–Meyer) test following the manufacturer’s instructions (Ward’s Science, Rochester, NY; Cox 1991).

The *Z*-score test was used to compare proportions of recovered, engorged populations. Relationships among measured Scutal Index factors were analyzed using Pearson’s correlation. Scutal Index differences between engorged and reference specimens and life stage were analyzed using analysis of variance (ANOVA) and Hedges’ g was used to measure corrected effect size for small sample sizes (Hedges 1981). Statistical analyses were performed using R v. 3.6.0 (R Core Team 2019) and the packages car (Fox and Weisberg 2019), effects (Fox 2003), plyr (Wickham 2011), and lsmeans (Lenth 2016).

## Results

During the surveillance period, a total of 2,267 unique sampling events were conducted yielding 6,922 *I. scapularis* (3,244 nymphs and 3,678 females) and 1,588 *H. longicornis* (1,425 nymphs and 163 females). Of these, 7 (0.22%) *I. scapularis* nymphs, 22 (1.54%) *H. longicornis* nymphs, and 5 (3.07%) *H. longicornis* females were engorged; no engorged *I. scapularis* females were detected. Significantly more partially engorged *H. longicornis* nymphs were recovered compared to *I. scapularis* nymphs (*Z* = −5.32; *P* < 0.001).

Other, nontarget ticks collected during surveillance were not engorged (*Amblyomma americanum* Linnaeus (Acari: Ixodidae) [*n* = 120], *Amblyomma maculatum* Koch (Acari: Ixodidae) [*n* = 60], *Dermacentor albipictus* Packard (Acari: Ixodidae) [*n* = 11], *Dermacentor variabilis* Say (Acari: Ixodidae) [*n* = 1,629], *Haemaphysalis leporispalustris* Packard (Acari: Ixodidae) [*n* = 1], *Ixodes dentatus* Marx (Acari: Ixodidae) [*n* = 2]).

For *I. scapularis* nymphs, body length (*F* = 0.71; df = 1, 18; *P* = 0.412) and scutum length (*F* = 4.24; df = 1, 18; *P* = 0.054) were not significantly different between microscopically determined unengorged field-collected specimens and unfed colony specimens. Similarly, Scutal Indices for unengorged, field-collected *H. longicornis* nymphs ($\bar{x}$= 2.58 ± 0.054) were comparable to unfed colony nymphs ($\bar{x}$= 2.56 ± 0.023). Given the similar measures between unfed field and colony ticks, we chose to continue engorgement comparisons using field-collected specimens to examine more relevant within-population variation.

Expectedly, Scutal Index measures (mean body and scutum lengths, Table 1) were highly correlated for both unengorged (*r* = 0.873; *P* = 0.001) and engorged (*r* = 0.870; *P* = 0.011) *I. scapularis* and unengorged (*r* = 0.988; *P* < 0.001) and engorged (*r* = 0.851; *P* < 0.001) *H. longicornis*.

**Table 1.** Mean (SE) scutal length, body length, Scutal Index for unengorged and engorged *Ixodes scapularis* and *Haemaphysalis longicornis* recovered during active surveillance in PA from 1 October 2020 to 31 August 2021

Species	Life stage	Engorgement level	*n*	Scutal length (mm)	Body Length (mm)	Scutal Index
*H. longicornis*	Adult	Unengorged	10	0.94 (0.030)	2.42 (0.057)	2.58 (0.054)
		Engorged	5	0.98 (0.023)	2.56 (0.080)	2.62 (0.116)
	Nymph	Unengorged	10	0.45 (0.009)	1.32 (0.020)	2.98 (0.059)
		Engorged	22	0.46 (0.013)	1.56 (0.061)	3.40 (0.132)
*I. scapularis*	Nymph	Unengorged	10	0.71 (0.014)	1.08 (0.023)	1.53 (0.016)
		Engorged	7	0.70 (0.016)	1.52 (0.055)	2.18 (0.043)

The two-way ANOVA indicated that *H. longicornis* Scutal Index varied significantly by life stage (*F* = 14.03; df = 1, 44; *P* < 0.001) and engorgement (*F* = 4.12; df = 1, 44; *P* = 0.048). Similarly, the one-way ANOVA indicated that *I. scapularis* Scutal Index varied significantly by engorgement (*F* = 253.0; df = 1, 15; *P* < 0.001).

Mean Scutal Index difference between unengorged and engorged nymph specimens was 0.65 and 0.42 for *I. scapularis* and *H. longicornis*, respectively (Table 1); standardized mean differences between unengorged and engorged *I. scapularis* (Hedges’ *g* = 7.84) was considerably greater than differences between unengorged and engorged *H. longicornis* (Hedges’ *g* = 0.79).

The phenolphthalein test produced a colorimetric reaction from only engorged specimens indicating a presumptive positive result for blood.

## Discussion

Since the establishment of *H. longicornis* in the United States, a growing body of research has examined their associated pathogens and vector competence (e.g., Breuner et al. 2020, Stanley et al. 2020, Tufts et al. 2021); however, fundamental determinants of their vectorial capacity (e.g., feeding habits and engorgement dynamics) remain relatively unknown. Here, we documented the recovery of partially engorged, questing *H. longicornis* ticks from active surveillance suggesting indirect evidence of interrupted feeding.

Given the slow process of blood digestion in ticks, the Scutal Index estimates are unlikely to have been appreciably affected by assimilatory processes (e.g., hemolysis, pinocytotic activity) (Akov 1982, Koh et al. 1991). Additionally, while residual host blood proteins may persist after tick feeding and molting, opisthosomal features indicatory of engorgement are likely indistinguishable (Wickramasekara et al. 2008). Therefore, engorgement measures herein are presumed representative of feeding events within stadia. Our results indicate that *H. longicornis* is significantly more likely to quest (within stadia) after taking a bloodmeal than *I. scapularis* and that those questing *H. longicornis* may have engorged to a lesser extent than *I. scapularis*.

Our *I. scapularis* findings support anecdotal observations from other *Ixodes* active surveillance efforts, suggesting rare occurrence of interrupted feedings or questing while engorged (van Duijvendijk et al. 2016). In contrast, a significantly greater abundance of engorged *H. longicornis* appear to quest, and engorgement measures suggests dislodgement from hosts prior to repletion. For instance, the mean Scutal Index difference between unengorged and engorged nymph specimens was 0.65 and 0.42 for *I. scapularis* and *H. longicornis*, respectively. This slight difference between *H. longicornis* specimens is suggestive of a smaller or disrupted bloodmeal. The difference in mean Scutal Index between unengorged and engorged female *H. longicornis* was particularly slight compared to the difference noted in nymphs. Copulation has been found to induce integument expansion in *H. longicornis* (Okura et al. 1996). Tick cuticle acidification specifically promotes this extensibility, and acidophilic epidermal cells, more sizeable and active in copulated female *H. longicornis*, are responsible for acidifying the cuticle (Okura et al. 1997, Kaufman et al. 2010). Copulatory stimuli would expectedly be absent in U.S. parthenogenetic populations (Egizi et al. 2020); and therefore, limited adult engorgement differences may also be a function of population dynamics. The broad variation recovered within mean Scutal Index for *H. longicornis* nymphs indicates that while they can feed to repletion, they appear to quest again after varied durations of host attachment (Falco et al. 1996). Recovery on drags nevertheless supports the limited work demonstrating reattachment competence of dislodged, partially engorged *Haemaphysalis* (Varma et al. 1960).

While it is difficult to speculate on the reason(s) why engorged ticks were recovered questing, 11 of 27 engorged *H. longicornis* were collected from the same park in Bucks County between Apr and Jun 2021, suggesting a potential spatial or environmental component. Perhaps a less preferred local host community composition could impede ticks feeding to repletion, as observed in laboratory-based studies (Ronai et al. 2020). Variable tick aggregation among local host species could also yield differential attachment/ engorgement success (Lydecker et al. 2019). A recent study of *H. longicornis* vertebrate host associations found other native ticks (*I. scapularis*) feeding in close proximity, suggesting they use the host landscape similarly and thus should not be disproportionately affected by host grooming (Tufts et al. 2021). However, anecdotal observations indicate that *H. longicornis* are relatively more sensitive to disturbance (Sherpa et al. 2021), and potentially more susceptible to dislodgment. Alternatively, particularly elevated *H. longicornis* infestation intensities and/ or diverse salivary components between *I. scapularis* and *H. longicornis* may elicit unique host immunomodulatory activities that contribute to differing blood-feeding success (Brossard and Wikel 2004, Kotsyfakis et al. 2007, Nuttall 2019, Tufts et al. 2021).

While the recovery of host-seeking, engorged *H. longicornis* offers ecological insights through questing and blood-feeding patterns and behaviors, these results may also have epidemiological significance. For instance, Bucks County, where most (17/27) of the partially engorged *H. longicornis* were recovered, is the same county where *Borrelia burgdorferi* sensu stricto Johnson, Schmid, Hyde, Steigerwalt & Brenner (Spirochaetales: Spirochaetaceae) DNA was detected in field-collected *H. longicornis* ticks (Price et al. 2021a). Even brief, interrupted blood feeding has been found sufficient for infection acquisition (Richter et al. 2012, Faulde et al. 2014). If partially engorged *H. longicornis* are infected from previous (within stadia) incomplete bloodmeal(s) and recurrently seek additional bloodmeals, this could decouple transstadial persistence conditions from vectorial capacity, especially for pathogens maintained in horizontal transmission cycles, e.g., *B. burgdorferi* (Eisen 2020). Additionally, partially fed ticks have been associated with accelerated pathogen transmission (Shih and Spielman 1993) and reports of *H. longicornis* biting humans are increasing (Bickerton and Toledo 2020, Lv et al. 2021). Collectively, these findings highlight the need for further tick and pathogen surveillance and characterization of *H. longicornis* engorgement to determine the extent to which partial feeding may moderate transstadial maintenance preconditions in vector competency studies (e.g., Breuner et al. 2020, Levin et al. 2021), increase vectorial capacity, and support tick-borne pathogen transmission (Davies 1990, Wang et al. 1999).

Overall, these data are among the first to document questing behavior of blooded *H. longicornis* ticks and provide initial field evidence for interrupted feeding and repeated bloodmeal quests (Tahir et al. 2020). Moreover, given the proportionately high recovery of engorged *H. longicornis*, apparent reattachment capability, and pathogen presence in other field-collected specimens during preceding surveillances, implications for pathogen transmission exists and warrant consideration in existing vector biology models and infection risk studies. Continued monitoring and documentation of this unique biological phenomenon is important, especially as populations continue to spread and establish. Future work aims to accrue molecular support for interrupted feeding in *H. longicornis* through host bloodmeal(s) identification (Tahir et al. 2020).

## Supplementary Data

Supplementary data are available at *Journal of Medical Entomology* online.

Supplementary Fig. 1. Images of unengorged (left) and engorged (right) *Haemaphysalis longicornis* nymphs recovered from active surveillance in Pennsylvania examined under 10x magnification and measured for total body length and scutal length to estimate engorgement (Scutal) index.

Supplementary Table 1. Total *Haemaphysalis longicornis* ticks recovered by life stage across surveillance period. Note, weather and climatic conditions precluded active surveillance operations in Feb 2021.

tjac099_suppl_Supplementary_Materials
