# Supplementary material for: Recovery of Partially Engorged Haemaphysalis longicornis (Acari: Ixodidae) Ticks from Active Surveillance
Source: J Med Entomol. 2022 Jul 19;59(5):1842–6. doi: 10.1093/jme/tjac099 (PMC9473650; doi:10.1093/jme/tjac099)

Supplemental Table 1. Total *Haemaphysalis longicornis* ticks recovered by life stage across surveillance period. Note, weather and climatic conditions precluded active surveillance operations in Feb 2021.

| Month/ Year | Larvae | Nymph | Adult (F) |
| --- | --- | --- | --- |
| Oct 2020 | 1453 | 4 | 0 |
| Nov 2020 | 38 | 1 | 0 |
| Dec 2020 | 0 | 0 | 0 |
| Jan 2021 | 0 | 0 | 0 |
| Feb 2021 | NA | NA | NA |
| Mar 2021 | 0 | 61 | 0 |
| Apr 2021 | 18 | 139 | 2 |
| May 2021 | 326 | 761 | 14 |
| Jun 2021 | 77 | 311 | 29 |
| Jul 2021 | 81 | 114 | 57 |
| Aug 2021 | 539 | 34 | 61 |


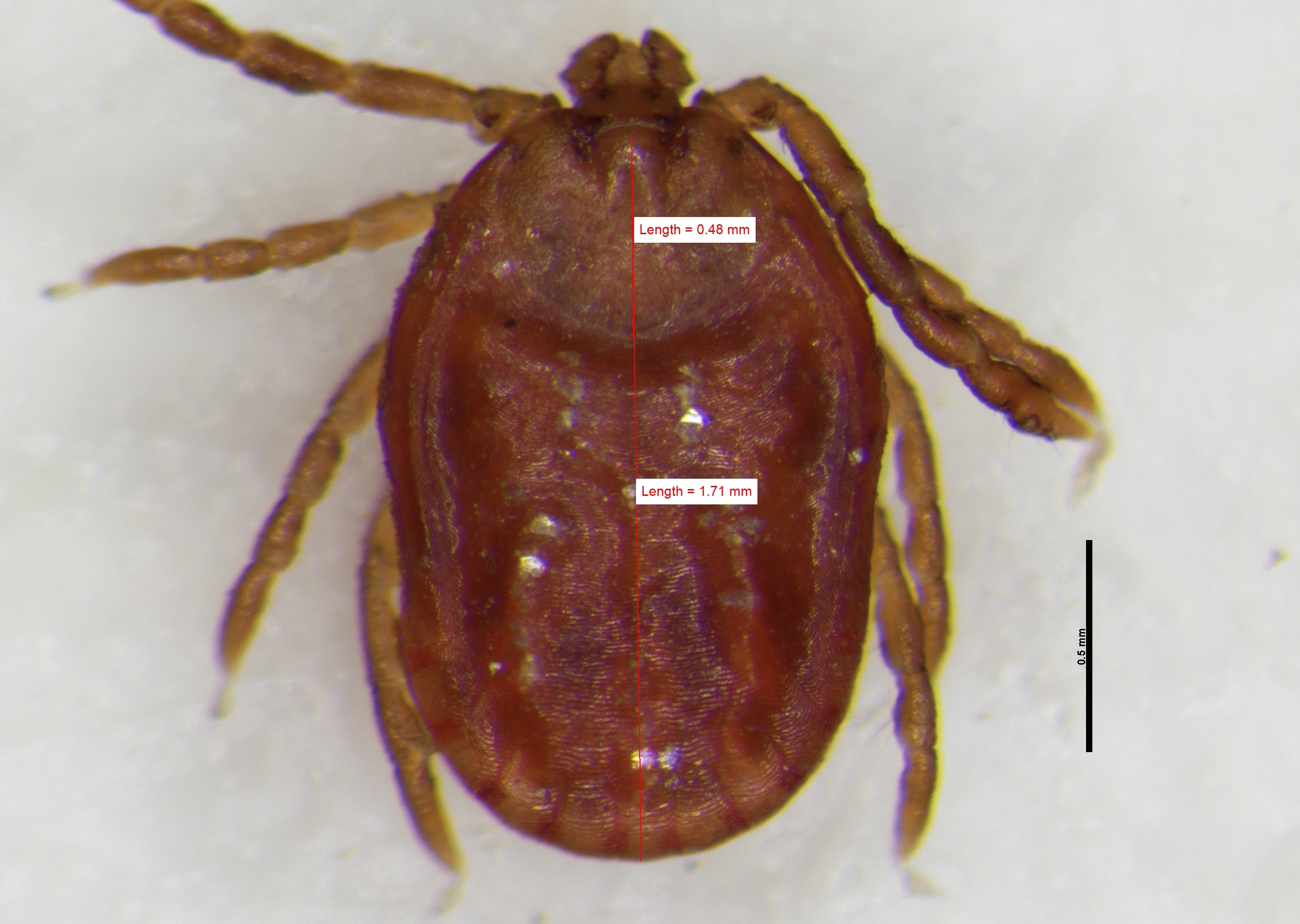

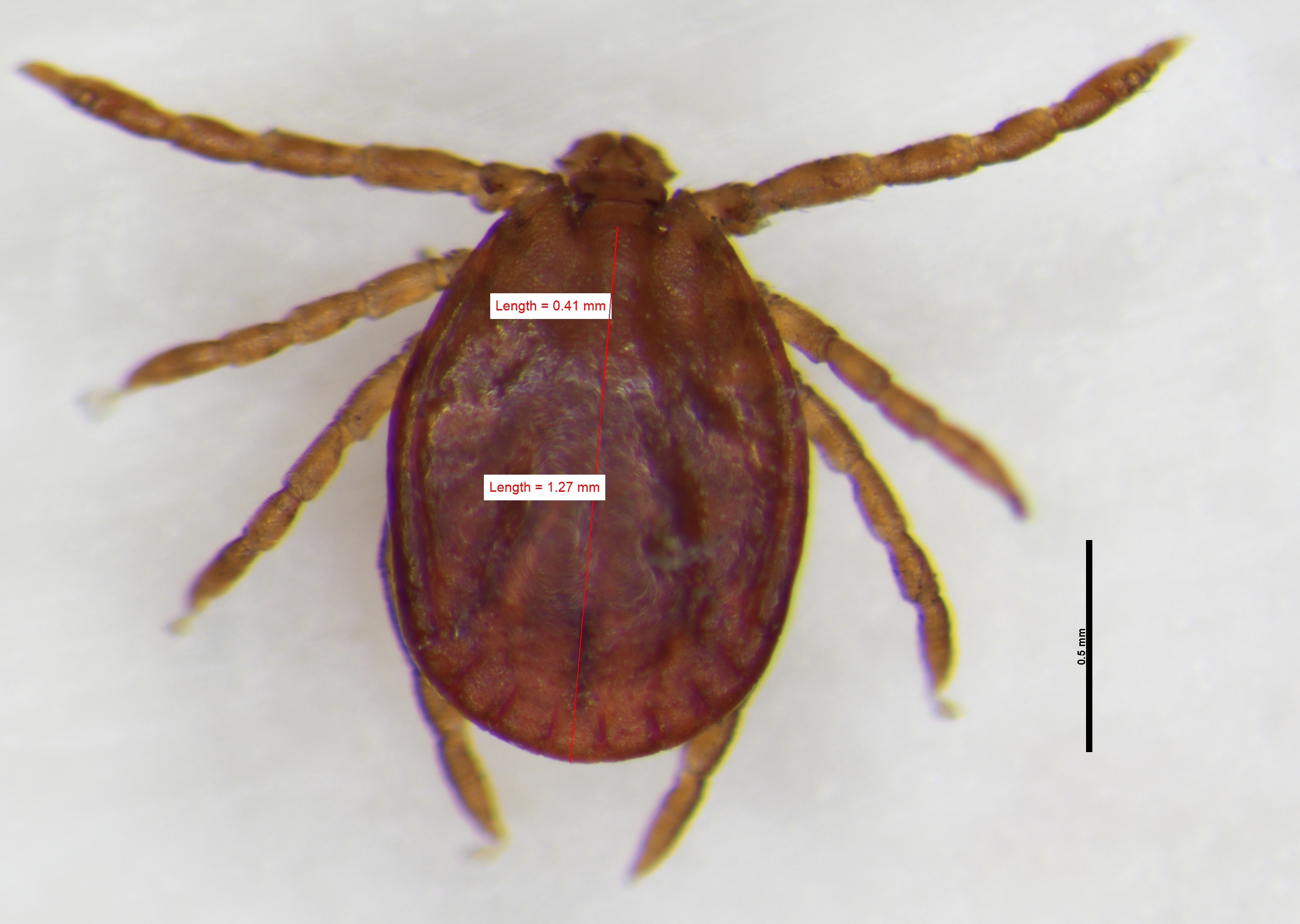

Supplement: tjac099_suppl_Supplementary_Materials [file tjac099_suppl_supplementary_materials.docx]
